# Reduced expression of mitochondrial fumarate hydratase in progressive multiple sclerosis contributes to impaired in vitro mesenchymal stromal cell-mediated neuroprotection

**DOI:** 10.1177/13524585211060686

**Published:** 2021-11-29

**Authors:** Pamela Sarkar, Juliana Redondo, Kelly Hares, Steven Bailey, Anastasia Georgievskaya, Kate Heesom, Kevin C Kemp, Neil J Scolding, Claire M Rice

**Affiliations:** Clinical Neurosciences, Translational Health Sciences, Bristol Medical School, University of Bristol, Bristol, UK; Department of Neurology, North Bristol NHS Trust, Southmead Hospital, Bristol, UK; Clinical Neurosciences, Translational Health Sciences, Bristol Medical School, University of Bristol, Bristol, UK; Clinical Neurosciences, Translational Health Sciences, Bristol Medical School, University of Bristol, Bristol, UK; Clinical Neurosciences, Translational Health Sciences, Bristol Medical School, University of Bristol, Bristol, UK; Department of Neurology, North Bristol NHS Trust, Southmead Hospital, Bristol, UK; Clinical Neurosciences, Translational Health Sciences, Bristol Medical School, University of Bristol, Bristol, UK; Bristol Proteomics Facility, Biomedical Sciences, University of Bristol, Bristol, UK; Clinical Neurosciences, Translational Health Sciences, Bristol Medical School, University of Bristol, Bristol, UK; Clinical Neurosciences, Translational Health Sciences, Bristol Medical School, University of Bristol, Bristol, UK; Department of Neurology, North Bristol NHS Trust, Southmead Hospital, Bristol, UK; Clinical Neurosciences, Translational Health Sciences, Bristol Medical School, University of Bristol, Bristol, UK; Department of Neurology, North Bristol NHS Trust, Southmead Hospital, Bristol, UK

**Keywords:** Multiple sclerosis, fumarate hydratase, mesenchymal stromal cells, neuroprotection, oxidative stress

## Abstract

**Background:**

Cell-based therapies for multiple sclerosis (MS), including those employing autologous bone marrow-derived mesenchymal stromal cells (MSC) are being examined in clinical trials. However, recent studies have identified abnormalities in the MS bone marrow microenvironment.

**Objective:**

We aimed to compare the secretome of MSC isolated from control subjects (C-MSC) and people with MS (MS-MSC) and explore the functional relevance of findings.

**Methods:**

We employed high throughput proteomic analysis, enzyme-linked immunosorbent assays and immunoblotting, as well as in vitro assays of enzyme activity and neuroprotection.

**Results:**

We demonstrated that, in progressive MS, the MSC secretome has lower levels of mitochondrial fumarate hydratase (mFH). Exogenous mFH restores the in vitro neuroprotective potential of MS-MSC. Furthermore, MS-MSC expresses reduced levels of fumarate hydratase (FH) with downstream reduction in expression of master regulators of oxidative stress.

**Conclusions:**

Our findings are further evidence of dysregulation of the bone marrow microenvironment in progressive MS with respect to anti-oxidative capacity and immunoregulatory potential. Given the clinical utility of the fumaric acid ester dimethyl fumarate in relapsing–remitting MS, our findings have potential implication for understanding MS pathophysiology and personalised therapeutic intervention.

## Introduction

Cell-based therapy for the treatment of multiple sclerosis (MS) has undergone rapid translation from in vitro and in vivo studies to clinical trials. In neurological disease, including MS, the potential of autologous cells isolated from a systemic source and expanded ex vivo is particularly attractive given the limited capacity of the central nervous system (CNS) for repair.^1^ The protective properties of multipotent mesenchymal stromal cells (MSC) and their secretome, in both in vitro and in vivo models of neurodegenerative disease, mean they are widely regarded as one of the most promising cell types for use in cell-based therapies.^2–5^

If autologous cells are to be employed in cell-based therapies, it is important to demonstrate that their therapeutic properties have not been compromised by exposure to disease.^6^ In MS, there is increasing concern that MSC isolated from people with MS have altered functional properties. We have previously demonstrated that MSC isolated from people with progressive with MS (MS-MSC) can be expanded in vitro and have the expected cell surface phenotype and mesenchymal differentiation potential.^7^ However, in subsequent, larger studies which take the effect of age into consideration, we demonstrated that MS-MSC have reduced ex vivo expansion potential,^8^ and failure or in adequate ex vivo expansion of autologous MSC was also reported in approximately 5% participants in the MEsenchymal StEm cells for MS (MESEMS) study (NCT01854957; A. Uccelli, ECTRIMS 2018).^9^ Our previous investigations have also demonstrated that MS-MSC have an in vitro phenotype consistent with premature ageing, with increased expression of markers of senescence and accelerated telomere shortening.^8^ Furthermore, we have also shown that the MS-MSC secretome offers reduced neuroprotection in vitro,^10^ and MS-MSC have increased susceptibility to nitrosative stress and display dysregulated anti-oxidant responses including reduced secretion of a range of trophic factors and anti-oxidants.^11^ Others have reported reduced immunosuppressive function and altered cytokine expression in MS-MSC^12,13^ as well as reduced therapeutic efficacy of MS-MSC in a murine model of MS (experimental autoimmune encephalomyelitis, EAE).^14^

Here, we compared the composition of the MSC secretome when MSC were isolated from people with MS or control subjects with the aim of identifying differences which may contribute to the reduced neuroprotective potential of the MSC secretome and dysregulated anti-oxidant responses previously reported.

## Material and methods

### MSC isolation and culture

Bone marrow aspirates were obtained from people with progressive MS (MS-MSC) participating in the clinical trials ‘Repeat Infusion of Autologous bone Marrow Cells in MS (SIAMMS-II)’ (NCT01932593; United Kingdom (UK) Research Ethics Committee (REC) 13/SW/0255)^15^ and ‘Assessment of Bone Marrow-Derived Cellular Therapy in Progressive Multiple Sclerosis (ACTiMuS)’ (NCT01815632; REC 12/SW/0358).^16^ Control MSC (C-MSC) were obtained from the discarded femoral head during total hip replacement (REC 10/H102/69); donors were known to have osteoarthritis, but were otherwise healthy and were not receiving drugs associated with myelosuppression. None of the ACTiMuS participants had received disease modifying therapy in the year prior to bone marrow collection although some participants with secondary progressive MS had been exposed to disease modifying therapy previously (see Supplementary Material). Not all samples were available for all experiments; the number of biological replicates is specified in each experiment and details regarding the cohort and which samples were used for each analysis are presented as Supplementary Material.

### Isolation of MSC and preparation of MSC-conditioned medium

MSC were isolated using a density gradient and were expanded in vitro as previously described.^17^ Cell surface phenotype and mesenchymal differentiation potential were confirmed to be consistent with those expected of MSC.^7^ MSC in the logarithmic phase of growth at second (p2) or third passage (p3) were used to produce conditioned medium.^17^ The culture flasks were washed twice with Dulbecco’s modified Eagle’s medium (DMEM; Sigma, USA), to remove any residual trophic effect from serum. Minimal medium (MIN) was added to the flasks. This consisted of 48.25 mL DMEM, 500 µL Pen-Strep (Gibco Penicillin–Streptomycin, Ref 15140-122), 500 µL Sato concentrate (containing 100 µg/mL of bovine serum albumin, 0.06 µg/mL progesterone, 16 µg/mL putrescine, 0.04 µg/mL selenite, 0.04 µg/mL thyroxine and 0.04 µg/mL triiodothyronine),^18^ 500 µL holo-transferrin (Sigma-Aldrich, Ref. T0665) and 250 µL L-glutamine (Sigma Aldrich, Ref. I5500). After 24 hours, conditioned medium was collected from cultures of control MSC (C-MSCcm) or MSC isolated from patients with progressive MS (MS-MSCcm), centrifuged, filtered and stored at −20°C.^17^

### Isolation of mitochondria

MSC mitochondria were isolated with a commercial kit used according to manufacturer’s instructions (Sigma MITOISO2). Briefly, cells at p3 were detached, washed in ice-cold phosphate-buffered saline (PBS) and lysed on ice for 5 minutes. Extraction buffer was added, and cells centrifuged at 600 × *g* for 10 minutes. Supernatant was collected and centrifuged at 11,000 × *g* for 10 minutes to obtain mitochondrial pellet. The pellet was resuspended in either storage buffer for mitochondrial activity assay or CelLytic M cell lysis reagent with protease inhibitors for immunoblotting.

### Proteomics

At the University of Bristol Proteomics Facility, liquid chromatography–tandem mass spectrometry (LC-MSMS) of C-MSCcm and MS-MSCcm was performed according to a previously described protocol for tandem mass tagging (Thermo Fisher Scientific, USA).^19^

### Enzyme-linked immunosorbent assay

Ready to use sandwich enzyme-linked immunosorbent assay (ELISA) for mFH (human mFH: Cusabio Catalogue No. CSB-EL008659HU) was performed on conditioned medium from C-MSC and MS-MSC according to the manufacturer’s instructions. A standard curve was prepared and absorbance read on a spectrophotometer at 450 nm (BMG Labtech Fluostar Optima). Values were interpolated into the curve and multiplied by the dilution factor to obtain the final concentration.

### Fumarase activity assay

Fumarase activity was quantified using a commercially available assay according to the manufacturer’s instructions (Sigma-Aldrich, Ref. MAK206). In addition to MSC or mitochondrial lysate, wells contained 50 µL reaction mix which consisted of 36 µL of fumarase assay buffer, 2 µL of fumarase enzyme mix, 10 µL of fumarase developer and 2 µL of fumarase substrate. After adding the reaction mix, the plate was protected from light and mixed using a horizontal shaker. The results were measured using a BMG Labtech Fluostar Optima microplate reader at 450 nm, and MARS data analysis software (kinetic mode for 60 minutes at 37°C with absorbance readings taken every minute). Nicotinamide adenine dinucleotide and hydrogen (NADH) standards were read at the end of the incubation time. To calculate fumarase activity, the absorbance for each well was plotted versus time. Two time points were chosen (T1 and T2) in the linear range of the plot, and the absorbance was determined. Background was corrected by subtracting the measurement obtained for the blank standards. The change in absorbance from T1 to T2 was calculated, and the amount of NADH generated (nmole/well) was obtained. Fumarase activity was ascertained by dividing amount of NADH (nmole) between T1 and T2 by the reaction time multiplied by sample volume added to the well, and the activity was reported as nmole/min/µL or milliunits/µL where one unit of fumarase is the amount of enzyme that generates 1.0 µmole of NADH per minute at pH 9.5 and 37°C.

### Immunoblotting

Immunoblotting was performed as previously described.^11,20^ Briefly, MSC were plated at 5 × 10^4^ cells per well in a six-well plate prior to lysis with universal lysis buffer (Millipore). Protein quantification was performed with Qubit Fluorometer and Quant-iT™ protein assay kit (Invitrogen) to ensure equal loading of samples. Protein lysates were diluted 1:1 with 2 × Laemmli buffer and denatured at 95°C, before loading on Tris HCl 4–20% ready gels (Bio-Rad). Gels were transferred to nitrocellulose membrane and subsequently blocked with 5% bovine serum albumin (Sigma) or 5% milk in tris-buffered saline–Tween for 1 hour. Incubation with primary antibody anti-FH (Abcam, ab95950), anti-nuclear-related (erythroid-derived 2) factor 2 (anti-NRF-2) antibody (R&D, AF3925), anti-hypoxia inducible factor1α (anti-hypoxia inducible factor1α (anti-HIF-1α; Abcam, ab51608)), anti-COX5 (Santa Cruz, SC-376907) and anti-glyceraldehyde 3-phosphate dehydrogenase (GAPDH; Abcam, ab9484) was performed overnight at 4°C. Amersham ECL Plus™ Western Blotting Detection System (GE Healthcare) was used to visualise specific protein expression patterns by chemiluminescence. The integrated density of bands was measured using ImageJ (National Institute of Health, NIH), and values are expressed relative to GAPDH loading control protein.

### Neurotoxicity assays

Rodent cortical neuronal cultures, trophic factor withdrawal (exposure to MIN for 24 hours) and nitric oxide (NO) toxicity assays employing 0.4 mM DETANONOate (Enzo Life Sciences) were established as previously described.^10,20^ Survival was assessed using the 3-(4,5-dimethylthiazol-2-yl)-2,5-diphenyltetrazolium bromide (MTT) assay.^21^ Recombinant human FH was added as described in the relevant experiments (Sigma-Aldrich, Ref. SRP6120).

### Statistical analysis

GraphPad PRISM 5 (GraphPad Software) was used for graphical illustrations and statistical analyses not employing multiple regression (*). Where stated, multivariant analyses (^#^) were performed with STATA v12 (StataCorp).^8,10,11^ Bar graphs show mean ± standard error of the mean and regression lines were fitted with 95% confidence intervals (CI). Values of *p* < 0.05 were considered statistically significant.

## Results

### Reduced mFH secretion in progressive MS is negatively associated with duration of progressive phase of disease

**Figure 1.**
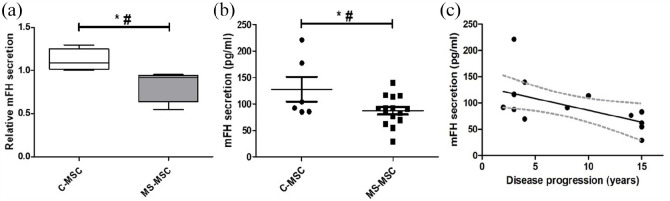
MS-MSC secrete reduced mFH and secretion is negatively associated with duration of progression in MS. (a) Secretion of mFH in C-MSC and MS-MSC as determined by LC-MSMS. (b) Quantitative determination of mFH secretion in MSCs as measured by ELISA. (c) Negative association of mFH secretion with duration of progressive phase of MS. mFH: mitochondrial fumarate hydratase; C-MSC: control mesenchymal stromal cells; MS-MSC: multiple sclerosis mesenchymal stromal cells. *p < 0.05, ^#^*p* < 0.05 multivariant analysis.

Relative reduction in the concentration of mFH in the secretome of MS-MSC was demonstrated by LC-MSMS (C-MSC: *n* = 4, MS-MSC: *n* = 4; *p = 0.048) and reduced concentration was confirmed by ELISA (C-MSC: *n* = 6, MS-MSC: *n* = 15; *p = 0.042; Figure 1). Following analysis of the quantitative ELISA data using the regression model to account for effects of age, a statistically significant independent effect of progressive MS was seen (^#^*p* = 0.041, CI = −4.614 to −0.097) and there was a negative association with duration of progressive phase of MS (Figure 1; Pearson’s *r* −0.568, CI = −0.837 to −0.079, *p = 0.027).

### FH activity in MS-MSC is preserved when adjusted for age

**Figure 2.**
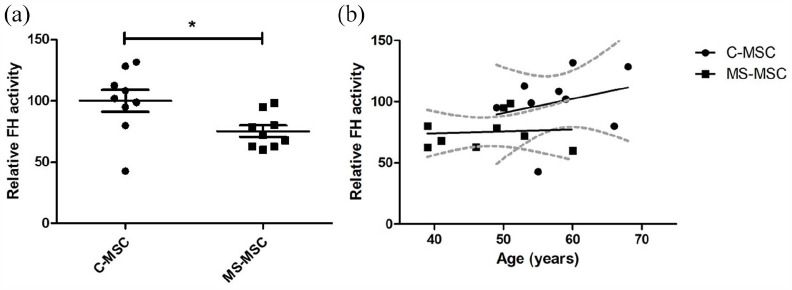
MS-MSC have reduced endogenous FH activity, but effect is confounded by differences in age between cohorts. (a) MS-MSC FH activity was reduced in MS-MSC compared with C-MSC although the observed effect does not persist following adjustment for age. (b) There was a strong trend towards an increase in FH activity with increasing age, although this effect was not seen in MS-MSC. FH: fumarate hydratase; C-MSC: control mesenchymal stromal cells; MS-MSC: multiple sclerosis mesenchymal stromal cells. *p < 0.05.

Under basal cell culture conditions, total fumarate hydratase (FH) activity was reduced in MS-MSC (*n* = 9) compared with C-MSC (*n* = 9; *p = 0.026; Figure 2). However, a statistically significant effect was not observed when confounding effects of age were taken into account; there was a strong trend towards an increase in FH activity with age (*p* = 0.059) although the trend was less marked in MS-MSC (Figure 2(b)). There was no association between FH activity in MS-MSC with duration of disease progression.

**Figure 3.**
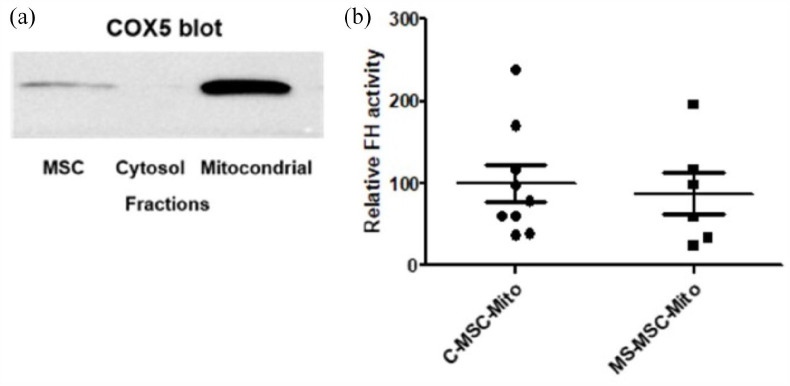
mFH activity is preserved in MS-MSC. (a) Immunoblot for COX5 confirms the specificity of MSC mitochondrial fractionation. (b) No difference in mFH activity was seen in mitochondria isolated from C-MSC and MS-MSC. MSC: mesenchymal stromal cells; COX5: cytochrome c oxidase subunit 5; mFH: mitochondrial fumarate hydratase; C-MSC-Mito: mitochondria isolated from control mesenchymal stromal cells; MS-MSC-Mito: mitochondria isolated from multiple sclerosis mesenchymal stromal cells.

A specific assay for mFH was not available, so that the FH activity assay was used to determine FH activity in mitochondrial cell preparations isolated from C-MSC and MS-MSC. To confirm successful fractionation of the cell preparations, immunoblotting for COX5 was undertaken with equal loading (20 µg) of mitochondrial protein, the cytosolic fraction (negative control) and unfractionated MSC protein. As expected, COX5 expression was greater in the mitochondrial fraction than in the unfractionated MSC lysate and was not detected in the cytosolic fraction (Figure 3(a)). There was no significant difference in FH activity in mitochondria isolated from MS-MSC (*n* = 6) compared to those from C-MSC (*n* = 9; *p* = 0.73; Figure 3(b)).

### Reduced expression of FH in MS-MSC

**Figure 4.**
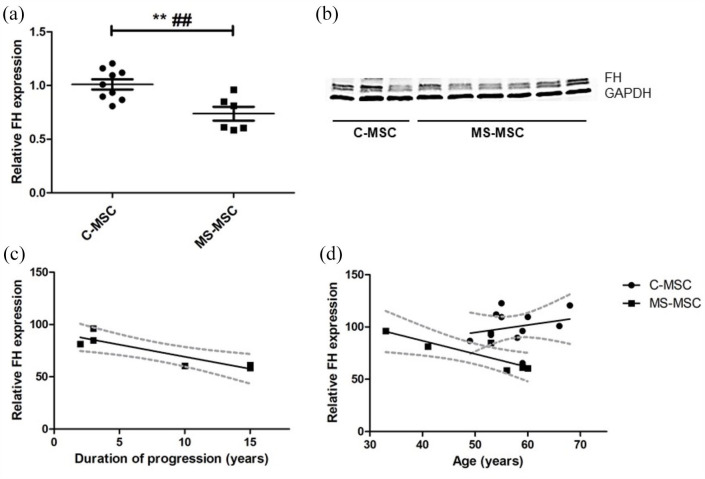
Reduced expression of FH protein by MS-MSC. (a) Reduced expression of FH protein was seen in MS-MSC and this effect persisted after adjustment for age difference between the cohorts. (b) Representative immunoblotting bands. (c) A reduction in relative FH expression was seen with increasing duration of disease progression in MS, but this effect was not statistically significant following adjustment for age (Pearson’s *r* = −0.897, *p* = 0.02, CI = −0.9888 to −0.3138; *p* > 0.05 following adjustment for age). (d) A differential effect of age on FH protein expression with age was seen between the cohorts; there was a significant negative association in MS-MSC and a strong trend towards a positive association in C-MSC. FH: fumarate hydratase; GAPDH: glyceraldehyde 3-phosphate dehydrogenase; C-MSC: control mesenchymal stromal cells; MS-MSC: multiple sclerosis mesenchymal stromal cells. **p < 0.01, ^##^*p* < 0.01 multivariant analysis.

Comparison of total FH expression by C-MSC and MS-MSC was examined by immunoblotting. Western blot analysis of MSC isolated from patients with MS (*n* = 6) and control subjects (*n* = 12) demonstrated reduced expression of FH protein by MS-MSC (**p = 0.004). This effect remained following adjustment with multiple regression for age (^##^*p* = 0.002, CI = −0.4824343 to −0.1456443; Figure 4(a) and (b)). There was a negative association between FH expression and increasing duration of progression of MS (Pearson’s *r* = −0.897, *p* = 0.02, CI = −0.9888 to −0.3138; Figure 4(c)), but this effect did not persist following adjustment for age. In the combined cohorts, there was no significant association between FH protein expression and age although a negative effect of increasing age was seen in MS-MSC (Pearson’s *r* = −0.872, *p* = 0.024, CI = −0.9859 to −0.2057). A trend towards a differential effect of age on FH was seen depending on the presence of progressive MS (*p* = 0.069) with a decrease in FH expression with age being seen only in MS-MSC (Figure 4(d)).

### Addition of FH to MS-MSCcm prevents neuronal loss under conditions of trophic factor withdrawal and nitrosative stress

We have previously demonstrated reduced neuronal survival in the presence of MS-MSCcm following trophic factor withdrawal and under conditions of nitrosative stress.^9^ Given the observed reduction in FH concentration in MS-MSCcm, we examined whether neuronal loss could be ameliorated by supplementation of MS-MSCcm with FH. Optimum concentration of exogenous FH was determined by a dose response curve which indicated that maximum neuroprotection was observed at concentrations of FH between 500 and 700 pg/mL, and replicates were performed with 500 pg/mL FH.

**Figure 5.**
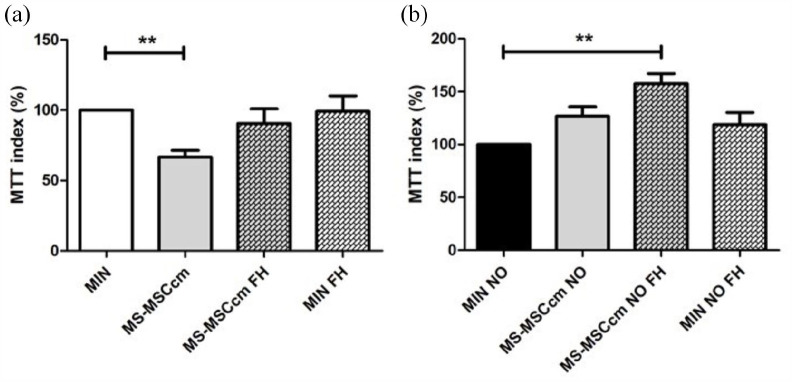
FH supplementation of MS-MSCcm restores neuroprotective potential of MS-MSCcm. (a) Under conditions of trophic factor withdrawal, reduced neuronal survival is observed in the presence of MS-MSCcm (*n* = 8) compared to minimal media (Kruskal–Wallis with Dunn’s multiple comparison test). However, with administration of exogenous FH to MS-MSCcm, neuronal loss is not observed. (b) Nitrosative stress was induced by application of DETANONOate (NO) and a protective effect of MS-MSCcm (*n* = 8) was seen only in the presence of exogenous FH (Kruskal–Wallis with Dunn’s multiple comparison test). FH: fumarate hydratase; C-MSCcm: conditioned medium from control mesenchymal stromal cells; MS-MSCcm: conditioned medium from multiple sclerosis mesenchymal stromal cells; MIN: minimal medium; MTT: 3-(4,5-dimethylthiazol-2-yl)-2,5-diphenyltetrazolium bromide; NO: nitric oxide; NS: not significant. **p < 0.01.

Following supplementation of MS-MSCcm with FH, neuronal loss was not observed under conditions of trophic factor withdrawal (Figure 5(a)) or nitrosative stress induced by addition of DETANONOate (Figure 5(b)).

### Nrf-2 expression is negatively associated with duration of progression in MS

High levels of intracellular fumarate have been associated with a range of downstream effects with potential implication for intracellular metabolic signalling. To begin to explore these, we examined expression of HIF-1α and Nrf-2; upregulation of both has been associated with loss of FH function^22,23^ and each has been identified as being of potential importance in the pathophysiology of MS.^24,25^

**Figure 6.**
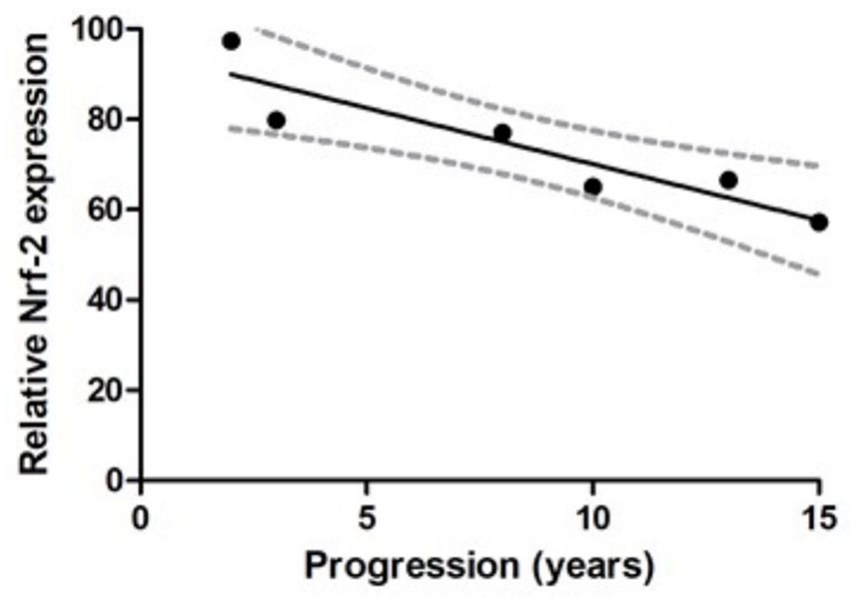
Nrf-2 protein expression. Reduced expression of Nrf-2 protein expression in association with increasing duration of disease progression in MS (Pearson’s *r* = −0.9819, *p* = 0.01, CI = −0.991 to −0.42). Nrf-2: nuclear-related (erythroid-derived 2) factor 2.

We have previously demonstrated that, although there is no difference in *Nrf-2* expression between C-MSC and MS-MSC, MS-MSC have reduced expression of Nrf-2 protein under standard culture conditions and in response to nitrosative stress.^11^ Here, we demonstrated a negative association between Nrf-2 protein expression and duration of disease progression in MS (*n* = 6, Pearson’s *r* = −0.9819, *p* = 0.01, CI = −0.991 to −0.42; Figure 6). An independent effect of age was not observed.

### Reduced HIF-1α expression in MS-MSC

**Figure 7.**
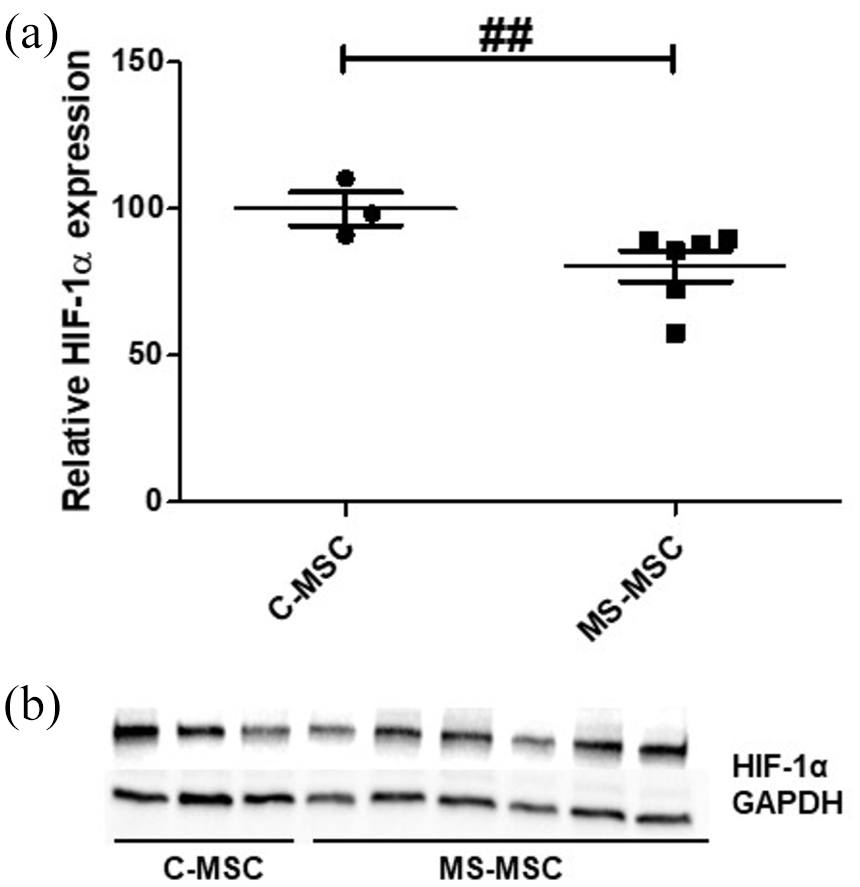
Reduced MS-MSC expression of HIF-1α. (a) HIF-1α protein expression is reduced in MS-MSC when the difference in age between the cohorts is accounted for. (b) Representative immunoblot. GAPDH: glyceraldehyde 3-phosphate dehydrogenase; HIF-1α: anti-hypoxia inducible factor1α; C-MSC: control mesenchymal stromal cells; MS-MSC: multiple sclerosis mesenchymal stromal cells. ^##^*p* < 0.01 multivariant analyses.

A strong trend towards reduced expression of HIF-1α protein in MS-MSC was noted on immunoblotting (C-MSC: *n* = 3, MS-MSC: *n* = 6; *p* = 0.056), reaching statistical significance when the effect of age was taken into account (^##^*p* = 0.001, CI = −0.4105565 to −0.1046486; Figure 7(a)). An independent effect of duration of progression was not observed.

## Discussion

To investigate the reduced neuroprotective potential of MS-MSC in vitro, we examined the MS-MSC secretome using LC-MSMS and noted reduced mFH secretion by MS-MSC. This was of particular interest given that dimethyl fumarate (DMF), a fumaric acid ester, is a licenced disease modifying therapy for relapsing–remitting MS and a putative neuroprotective effect has been reported.^26^ We confirmed reduced secretion of mFH by MS-MSC by ELISA, and there was a negative association with duration of progressive disease. Although reduced FH activity was observed in MS-MSC, this effect did not reach statistical significance after adjustment for differences in age between the cohorts and furthermore, no difference was seen when FH activity was assessed in mitochondrial preparations from MSC isolated from control subjects and people with progressive MS. However, reduced expression of FH was seen in MS-MSC and a negative correlation with duration of MS progression was observed. Exogenous application of FH was neuroprotective in vitro; neuronal survival with exposure to MS-MSCcm under conditions of trophic factor withdrawal and exposure to nitrosative stress increased. Although expression of both Nrf-2 and HIF-1α, downstream targets of FH, are both reduced in MS-MSC, only Nrf-2 expression negatively correlated with duration of progressive MS.

Fumarase deficiency (also known as fumaric aciduria) is a rare, life-limiting, autosomal recessive disorder associated with encephalopathy, hypotonia and seizures. Heterozygous germline mutations of *FH* are associated with hereditary leiomyomatosis and renal cell cancer (HLRCC). In addition to its role as a tumour suppressor, reduced expression of FH has been implicated in hypertension,^27^ type 2 diabetes^28^ and diabetic kidney disease.^29^ In mice, FH has been identified as a key regulator of metabolism in haematopoietic stem cells and deficiency is associated with aberrant lymphoid differentiation.^30^

In the Krebs cycle, fumarate is catalysed to malate by FH. Intracellular accumulation of fumarate in FH deficiency has a multitude of downstream metabolic consequences and the effects are known to vary according to cell type, but include increased oxidative stress and increased cellular senescence,^31^ mitochondrial dysfunction and activation of both the pro-oncogenic HIF and anti-oxidant Nrf-2 pathways.^32^ The latter are known to be of relevance to pathophysiology in MS^33,34^ and Nrf-2 has been proposed to underlie putative neuroprotective effects associated with DMF^32,35^ which is known to be of clinical benefit in relapsing–remitting MS.^36^ In our studies however, reduced mFH expression was associated with reduced Nrf-2 expression which may reflect a cell-specific or disease effect.

In many cell types, reduced expression of Nrf-2 is associated with increased HIF-1α and causes a shift to glycolysis. Although blocking aerobic glycolysis might be predicted to be anti-inflammatory,^37^ in MSC HIF-1α expression has been reported to promote MSC survival as well as maintenance of differentiation potential and MSC-mediated immunosuppression.^35^ Our finding of reduced HIF-1α expression in MS-MSC is therefore notable.

The current study suggests that FH deficiency in MS-MSC contributes to a dysfunctional bone marrow microenvironment in MS with potential significance for metabolic status and immunoregulation that warrants additional investigation to determine whether this is a disease-specific effect with potential for therapeutic intervention.

## Supplemental Material

sj-docx-1-msj-10.1177_13524585211060686 – Supplemental material for Reduced expression of mitochondrial fumarate hydratase in progressive multiple sclerosis contributes to impaired in vitro mesenchymal stromal cell-mediated neuroprotection Click here for additional data file. Supplemental material, sj-docx-1-msj-10.1177_13524585211060686 for Reduced expression of mitochondrial fumarate hydratase in progressive multiple sclerosis contributes to impaired in vitro mesenchymal stromal cell-mediated neuroprotection by Pamela Sarkar, Juliana Redondo, Kelly Hares, Steven Bailey, Anastasia Georgievskaya, Kate Heesom, Kevin C Kemp, Neil J Scolding and Claire M Rice in Multiple Sclerosis Journal

## References

[1] Ben-Hur T Fainstein N Nishri Y . Cell-based reparative therapies for multiple sclerosis. Curr Neurol Neurosci Rep 2013; 13(11): 397. 24078453 10.1007/s11910-013-0397-5

[2] Wilkins A Kemp K Ginty M , et al. Human bone marrow-derived mesenchymal stem cells secrete brain-derived neurotrophic factor which promotes neuronal survival in vitro. Stem Cell Res 2009; 3(1): 63–70. 19411199 10.1016/j.scr.2009.02.006

[3] Bai L Lennon DP Caplan AI , et al. Hepatocyte growth factor mediates mesenchymal stem cell–induced recovery in multiple sclerosis models. Nat Neurosci 2012; 15(6): 862–870. 22610068 10.1038/nn.3109 PMC3427471

[4] Bara JJ Turner S Roberts S , et al. High content and high throughput screening to assess the angiogenic and neurogenic actions of mesenchymal stem cells in vitro. Exp Cell Res 2015; 333: 93–104. 25678370 10.1016/j.yexcr.2014.12.019

[5] Mita T Furukawa-Hibi Y Takeuchi H , et al. Conditioned medium from the stem cells of human dental pulp improves cognitive function in a mouse model of Alzheimer’s disease. Behav Brain Res 2015; 293: 189–197. 26210934 10.1016/j.bbr.2015.07.043

[6] Munir H McGettrick HM . Mesenchymal stem cell therapy for autoimmune disease: Risks and rewards. Stem Cells Dev 2015; 24: 2091–2100. 26068030 10.1089/scd.2015.0008

[7] Mallam E Kemp K Wilkins A , et al. Characterization of in vitro expanded bone marrow-derived mesenchymal stem cells from patients with multiple sclerosis. Mult Scler 2010; 16(8): 909–918. 20542920 10.1177/1352458510371959

[8] Redondo J Sarkar P Kemp K , et al. Reduced cellularity of bone marrow in multiple sclerosis with decreased MSC expansion potential and premature ageing in vitro. Mult Scler 2018; 24(7): 919–931. 28548004 10.1177/1352458517711276 PMC6029147

[9] Uccelli A Laroni A Brundin L , et al. MEsenchymal StEm cells for Multiple Sclerosis (MESEMS): A randomized, double blind, cross-over phase I/II clinical trial with autologous mesenchymal stem cells for the therapy of multiple sclerosis. Trials 2019; 20: 263. 31072380 10.1186/s13063-019-3346-z PMC6507027

[10] Sarkar P Redondo J Kemp K , et al. Reduced neuroprotective potential of the mesenchymal stromal cell secretome with ex vivo expansion, age and progressive multiple sclerosis. Cytotherapy 2018; 20(1): 21–28. 28917625 10.1016/j.jcyt.2017.08.007 PMC5758344

[11] Redondo J Sarkar P Kemp K , et al. Dysregulation of mesenchymal stromal cell antioxidant responses in progressive multiple sclerosis. Stem Cells Transl Med 2018; 7(10): 748–758. 30063300 10.1002/sctm.18-0045 PMC6186266

[12] de Oliveira GL de Lima KW Colombini AM , et al. Bone marrow mesenchymal stromal cells isolated from multiple sclerosis patients have distinct gene expression profile and decreased suppressive function compared with healthy counterparts. Cell Transplant 2015; 24(2): 151–165. 24256874 10.3727/096368913X675142

[13] Mazzanti B Aldinucci A Biagioli T , et al. Differences in mesenchymal stem cell cytokine profiles between MS patients and healthy donors: Implication for assessment of disease activity and treatment. J Neuroimmunol 2008; 199: 142–150. 18562015 10.1016/j.jneuroim.2008.05.006

[14] Sargent A Bai L Shano G , et al. CNS disease diminishes the therapeutic functionality of bone marrow mesenchymal stem cells. Exp Neurol 2017; 295: 222–232. 28602834 10.1016/j.expneurol.2017.06.013 PMC5536847

[15] Rice CM Mallam EA Whone AL , et al. Safety and feasibility of autologous bone marrow cellular therapy in relapsing-progressive multiple sclerosis. Clin Pharmacol Ther 2010; 87: 679–685. 20445531 10.1038/clpt.2010.44

[16] Rice CMM Marks DII Ben-Shlomo Y , et al. Assessment of bone marrow-derived Cellular Therapy in progressive Multiple Sclerosis (ACTiMuS): Study protocol for a randomised controlled trial. Trials 2015; 16: 463. 26467901 10.1186/s13063-015-0953-1 PMC4606493

[17] Kemp K Hares K Mallam E , et al. Mesenchymal stem cell-secreted superoxide dismutase promotes cerebellar neuronal survival. J Neurochem 2010; 114(6): 1569–1580. 20028455 10.1111/j.1471-4159.2009.06553.x

[18] Bottenstein JE Sato GH . Growth of a rat neuroblastoma cell line in serum-free supplemented medium. Proc Natl Acad Sci USA 1979; 76(1): 514–517. 284369 10.1073/pnas.76.1.514 PMC382972

[19] Kittivorapart J Karamatic Crew V Wilson MC , et al. Quantitative proteomics of plasma vesicles identify novel biomarkers for hemoglobin E/b-thalassemic patients. Blood Adv 2018; 2: 95–104. 29365317 10.1182/bloodadvances.2017011726 PMC5787864

[20] Redondo J Hares K Wilkins A , et al. Reductions in kinesin expression are associated with nitric oxide-induced axonal damage. J Neurosci Res 2015; 93(6): 882–892. 25639260 10.1002/jnr.23556 PMC4513399

[21] Whone AL Kemp K Sun M , et al. Human bone marrow mesenchymal stem cells protect catecholaminergic and serotonergic neuronal perikarya and transporter function from oxidative stress by the secretion of glial-derived neurotrophic factor. Brain Res 2012; 1431: 86–96. 22143094 10.1016/j.brainres.2011.10.038

[22] Ooi A Wong J-C Petillo D , et al. An antioxidant response phenotype shared between hereditary and sporadic type 2 papillary renal cell carcinoma. Cancer Cell 2011; 20: 511–523. 22014576 10.1016/j.ccr.2011.08.024

[23] Isaacs JS Jung YJ Mole DR , et al. HIF overexpression correlates with biallelic loss of fumarate hydratase in renal cancer: Novel role of fumarate in regulation of HIF stability. Cancer Cell 2005; 8(2): 143–153. 16098467 10.1016/j.ccr.2005.06.017

[24] Asgari R Yarani R Mohammadi P , et al. HIF-1α in the crosstalk between reactive oxygen species and autophagy process: A review in multiple sclerosis. Cell Mol Neurobiol. Epub ahead of print 5 June 2021. DOI: 10.1007/s10571-021-01111-5. PMC11421632 34089426

[25] Michaličková D Hrnčíř T Canová NK , et al. Targeting Keap1/Nrf2/ARE signaling pathway in multiple sclerosis. Eur J Pharmacol 2020; 873: 172973. 32017935 10.1016/j.ejphar.2020.172973

[26] Linker RA Lee DH Ryan S , et al. Fumaric acid esters exert neuroprotective effects in neuroinflammation via activation of the Nrf2 antioxidant pathway. Brain 2011; 134(Pt 3): 678–692. 21354971 10.1093/brain/awq386

[27] Tian Z Liu Y Usa K , et al. Novel role of fumarate metabolism in Dahl-salt sensitive hypertension. Hypertension 2009; 54(2): 255–260. 19546378 10.1161/HYPERTENSIONAHA.109.129528 PMC2721687

[28] Adam J Ramracheya R Chibalina MV , et al. Fumarate hydratase deletion in pancreatic β cells leads to progressive diabetes. Cell Rep 2017; 20: 3135–3148. 28954230 10.1016/j.celrep.2017.08.093 PMC5637167

[29] You YH Quach T Saito R , et al. Metabolomics reveals a key role for fumarate in mediating the effects of NADPH oxidase 4 in diabetic kidney disease. J Am Soc Nephrol 2016; 27(2): 466–481. 26203118 10.1681/ASN.2015030302 PMC4731129

[30] Guitart AV Panagopoulou TI Villacreces A , et al. Fumarate hydratase is a critical metabolic regulator of hematopoietic stem cell functions. J Exp Med 2017; 214: 719–735. 28202494 10.1084/jem.20161087 PMC5339674

[31] Zheng L Cardaci S Jerby L , et al. Fumarate induces redox-dependent senescence by modifying glutathione metabolism. Nat Commun 2015; 6: 6001. 25613188 10.1038/ncomms7001 PMC4340546

[32] Yang M Soga T Pollard PJ , et al. The emerging role of fumarate as an oncometabolite. Front Oncol 2012; 2: 85. 22866264 10.3389/fonc.2012.00085 PMC3408580

[33] Buendia I Michalska P Navarro E , et al. Nrf2-ARE pathway: An emerging target against oxidative stress and neuroinflammation in neurodegenerative diseases. Pharmacol Ther 2016; 157: 84–104. 26617217 10.1016/j.pharmthera.2015.11.003

[34] Trapp BD Nave KA . Multiple sclerosis: An immune or neurodegenerative disorder? Annu Rev Neurosci 2008; 31: 247–269. 18558855 10.1146/annurev.neuro.30.051606.094313

[35] Contreras-Lopez R Elizondo-Vega R Paredes MJ , et al. HIF1α-dependent metabolic reprogramming governs mesenchymal stem/stromal cell immunoregulatory functions. FASEB J 2020; 34(6): 8250–8264. 32333618 10.1096/fj.201902232R

[36] Kappos L Gold R Miller DH , et al. Efficacy and safety of oral fumarate in patients with relapsing-remitting multiple sclerosis: A multicentre, randomised, double-blind, placebo-controlled phase IIb study. Lancet 2008; 372: 1463–1472. 18970976 10.1016/S0140-6736(08)61619-0

[37] Kornberg MD . The immunologic Warburg effect: Evidence and therapeutic opportunities in autoimmunity. Wiley Interdiscip Rev Syst Biol Med 2020; 12: e1486. 10.1002/wsbm.1486 PMC7507184 32105390

