# Supplementary material for: Reduced expression of mitochondrial fumarate hydratase in progressive multiple sclerosis contributes to impaired in vitro mesenchymal stromal cell-mediated neuroprotection
Source: Mult Scler. 2021 Nov 29;28(8):1179–88. doi: 10.1177/13524585211060686 (PMC9189727; doi:10.1177/13524585211060686)
Supplement: sj-docx-1-msj-10.1177_13524585211060686 – Supplemental material for Reduced expression of mitochondrial fumarate hydratase in progressive multiple sclerosis contributes to impaired in vitro mesenchymal stromal cell-mediated neuroprotection [file sj-docx-1-msj-10.1177_13524585211060686.docx]

**Table 1** Summary data for control and MS cohorts

| **n** | **Gender** | **Age (years)** | **Control/MS phenotype** | **EDSS** | **Duration progression (years)** | **Exposure to disease modifying treatment** | **Experiments performed** |
| --- | --- | --- | --- | --- | --- | --- | --- |
|  |  |  |  |  |  |  |  |
| 1 | M | 54 | Control | - | - | - | LC-MSMS; FH ELISA; FH activity; FH immunoblot |
| 2 | M | 66 | Control | - | - | - | LC-MSMS; FH activity; FH immunoblot; neurotrophic & toxicity assay |
| 3 | M | 49 | Control | - | - | - | FH activity; FH immunoblot |
| 4 | F | 60 | Control | - | - | - | FH ELISA; FH activity; FH immunoblot; neurotrophic & toxicity assay |
| 5 | M | N/A | Control | - | - | - | LC-MSMS |
| 6 | F | 68 | Control | - | - | - | LC-MSMS; FH activity; FH immunoblot; neurotrophic & toxicity assay |
| 7 | M | 58 | Control | - | - | - | FH ELISA; FH activity; neurotrophic & toxicity assay |
| 8 | F | 53 | Control | - | - | - | FH ELISA; FH activity; FH immunoblot |
| 9 | F | 55 | Control | - | - | - | FH ELISA; FH activity; FH immunoblot; neurotrophic & toxicity assay |
| 10 | M | 58 | Control | - | - | - | FH immunoblot |
| 11 | M | 59 | Control | - | - | - | FH ELISA; FH activity; FH immunoblot; neurotrophic & toxicity assay |
| 12 | F | 39 | Control | - | - | - | FH activity on mitochondrial preparation |
| 13 | M | 60 | Control | - | - | - | FH activity on mitochondrial preparation |
| 14 | M | 57 | Control | - | - | - | FH activity on mitochondrial preparation |
| 15 | F | 64 | Control | - | - | - | FH activity on mitochondrial preparation |
| 16 | F | 54 | Control | - | - | - | FH activity on mitochondrial preparation |
| 17 | F | 72 | Control | - | - | - | FH activity on mitochondrial preparation |
| 18 | F | 81 | Control | - | - | - | FH activity on mitochondrial preparation |
| 19 | M | 60 | Control | - | - | - | FH activity on mitochondrial preparation |
| 20 | F | 85 | Control | - | - | - | FH activity on mitochondrial preparation |
| 21 | M | 49 | SP | 4.5 | 4 | Nil | FH ELISA |
| 22 | M | 48 | PP | 6.0 | 4 | Nil | LC-MSMS |
| 23 | F | 48 | PP | 6.5 | 15 | Nil | LC-MSMS; FH ELISA |
| 24 | M | 60 | SP | 6.0 | 10 | Nil | FH ELISA; FH activity; FH immunoblot; Nrf2 |
| 25 | M | 33 | SP | 6.0 | 3 | Beta-interferon | LC-MSMS; FH ELISA; FH immunoblot |
| 26 | F | 47 | PP | 4.0 | 6 | Nil | FH ELISA |
| 27 | F | 59 | SP | 6.0 | 15 | Nil | LC-MSMS; FH immunoblot; Nrf2; HIF |
| 28 | M | 55 | SP | 4.0 | 2 | Nil | FH ELISA |
| 29 | F | 39 | SP | 6.0 | 8 | Nil | FH ELISA; FH activity; Nrf2; HIF |
| 30 | M | 56 | SP | 6.0 | 15 | Nil | FH ELISA; FH immunoblot; HIF |
| 31 | F | 53 | SP | 4.5 | 3 | Nil | FH ELISA; FH activity; HIF |
| 32 | M | 49 | PP | 6.0 | 14 | Nil | FH ELISA; neurotrophic & toxicity assay |
| 33 | M | 64 | PP | 6.0 | 15 | Nil | FH ELISA; neurotrophic & toxicity assay |
| 34 | M | 63 | SP | 6.0 | 12 | Beta-interferon | Nrf2; HIF |
| 35 | M | 59 | PP | 6.0 | 15 | Fampridine | FH ELISA |
| 36 | F | 41 | SP | 4.5 | 2 | Glatiramer; Beta-interferon | FH ELISA; FH activity; FH immunoblot; neurotrophic & toxicity assay; Nrf2; HIF |
| 37 | F | 50 | PP | 6.0 | 4 | Nil | FH ELISA; neurotrophic & toxicity assay |
| 38 | F | 49 | SP | 4.5 | 4 | Beta-interferon | FH ELISA; FH activity; neurotrophic & toxicity assay |
| 39 | F | 58 | PP | 5.5 | 10 | Nil | Neurotrophic & toxicity assay |
| 40 | F | 57 | SP | 5.5 | 3 | Glatiramer; Fampridine | FH immunoblot; neurotrophic & toxicity assay; Nrf2 |
| 41 | F | 54 | PP | 6.0 | 4 | Nil | Neurotrophic & toxicity assay |
| 42 | M | 53 | PP | 6.0 | 20 | Fampridine | FH activity |
| 43 | F | 52 | SP | 6.0 | 6 | Fampridine | FH activity |
| 44 | F | 47 | PP | 6.0 | 16 | Nil | FH activity |
| 45 | M | 58 | SP | 6.0 | 2 | Beta-interferon | FH activity on mitochondrial preparation |
| 46 | M | 56 | PP | 5.0 | 9 | Nil | FH activity on mitochondrial preparation |
| 47 | M | 58 | SP | 4.5 | 2 | Nil | FH activity on mitochondrial preparation |
| 48 | F | 52 | SP | 5.5 | 5 | Nil | FH activity on mitochondrial preparation |
| 49 | F | 58 | SP | 4.5 | 6 | Nil | FH activity on mitochondrial preparation |
| 50 | M | 61 | SP | 5.0 | 5 | Beta-interferon | FH activity on mitochondrial preparation |
| 51* | M | 57 | SP | 4.5 | 22 | Nil | LC-MSMS |
| 52* | F | 47 | SP | 6.5 | 16 | Nil | LC-MSMS |

The mean age for control subjects was 60.63 years (median 59 years). For people with MS, the mean age was years 52.81 years (median 53.5 years) (p=0.003). The mean EDSS was 5.47 (median 6).

ELISA enzyme-linked immunosorbent assay; EDSS Expanded Disability Status Scale; F female; FH fumarate hydratase; HIF hypoxia inducible factor1α; M male; LC-MSMS liquid chromatography-tandem mass spectrometry; N/A not available; Nrf2 nuclear related (erythroid derived 2)-factor 2; PP primary progressive; SP secondary progressive; * participant in ‘Study of Intravenous Autologous Marrow in Multiple Sclerosis’ (SIAMMS-II) study.
